# Infection from the Mouth

**Published:** 1894-04

**Authors:** E. L. Clifford

**Affiliations:** Chicago


### Infection from the Mouth.

Read in the Section on Dental and Oral Surgery, at the Forty-fourth Annual Meeting of the American Medical Association.

BY E. L. CLIFFORD, D.D.S., CHICAGO.

Each and every day brings the thoughtful student more and more to face the fact that the scientific world is progressing; and he who has kept *au courant* with the literature of medicine must acknowledge that the results of such investigations as are now being pursued have, for a principal object, the unfolding of the prime factors in the causation of diseases, with a view to their prevention.

Recent investigators have sought to open up new fields; they have tried, and found wanting, in many instances, the beaten track, and their efforts tend to the opening of new lines and a practical departure from the pathology of the past, with an ever increasing tendency to lead us to cultivate and entertain broader, or at any rate less narrow views, as to the elements which play an important part as etiologic factors in the causation and origin of diseases.

It is not necessary, or even advisable at this time, to recapitulate the theories of the past, and our aim will have been attained if we can take a few profitable glimpses at the newer fields, allowing the lines of thought and inquiry which may suggest themselves to each to furnish a basis for intelligent discussion.

Much has certainly been accomplished in the last two decades in the field of etiologic pathology, still much remains to be done, and upon the shoulders of some of the bright lights in our specialty and this Section will certainly fall a part of that task.

Investigation and experimentation in the past have been unable to specify and establish any *single* thing as a final cause of disease; and it has been said by one, eminent in our current literature, that he knew of no disease which acknowledged a *single* cause. Our work, then, if this be true, must be to search for and find out the many and ever varying factors or conditions which, as antecedents, combine to produce disease; and I am of the opinion that to the physiologic agencies within our bodies during life we will find many competent factors.

The researches and expositions of Gautier, Peter, Brown and Brunton, confirm our belief in the poisoning or intoxication of the animal economy with its own products. These eminent physiologic chemists traced and pursued their investigations from two general aspects, and Aitken in his excellent review of their work has classified them under two heads: 1, from a chemic and physiologic, or bio-chemic standpoint; and 2, from a clinical or pathologic. From the first standpoint the fact was established that in dead animal tissues, processes of putrefactive decompositions set in, by which certain alkaloids are elaborated from the proteid substances, and these alkaloids were designated by the late Selmi, of Bologna, as "ptomaines." Gautier, however, further showed that in the *living* animal tissues, and that by virtue of their vitality, certain other alkaloids are elaborated, which are analogous to the ptomaines, and these he has named "leucomaines." But, still further, in addition to these facts, he has demonstrated that in the living animal economy there are elaborated certain azotized uncrystallizable substances which are as yet undetermined, and which he has called "extractives" or "extractive matters," and which Aitken states are quite as unknown as the x, y, z's of an algebraic formula.

The nature of these extractives, then, remains a mystery, but this much we are told of them: that while we are assured that the ptomaines are toxic and that the leucomaines are also toxic, these unknown extractives are more toxic or poisonous to the system than either. Different alkaloids have been obtained from different sources, both animal and vegetable, and as long ago as 1820, Kerner pointed out the resemblance between the symptoms of poisoning by the animal and vegetable alkaloids. Experimentation since has confirmed the theory. "Zuelzer and Sonnenschein obtained, both from macerated dead bodies and from putrid meat infusions, small quantities of a crystallizable substance which exhibited the reactions of an alkaloid, and had a physiologic reaction like atropin, dilating the pupil, paralyzing the muscular fibers of the intestines and increasing the rapidity of the pulse." V. Anrep obtained an alkaloid from poisonous fish, and Vaughan an alkaloid from poisonous cheese, but Gautier, Etard, Brieger and others have given precision to the data previously acquired, and added largely to the varied and careful examinations of cadaveric tissues;" "they forced the conclusion that during putrefaction of nitrogenous animal material there are formed organic bases, fixed or volatile, presenting for their chemic and physiologic properties, the closest similitude to the vegetable alkaloids."

In fact, while it was at first supposed that these animal alkaloids differed in their nature from the organic alkaloids formed by vegetables, and various reactions had been given to distinguish them, Brieger appears to show that the distinction can be maintained no longer; but that the animal and the vegetable alkaloids are similar in their chemic constitution; that they are both products of albuminous proteid decompositions, and that some, at least, of the so-called ptomaines are identical with vegetable alkaloids. Hence, Dr. Brunton seems justified in regarding alkaloids "as products of albuminous decomposition, whether their albuminous precursor be contained in the cells of plants and altered during the process of growth, or whether the albuminous substances undergo decomposition outside or inside the animal body, or by processes of digestion, as by organized ferments." Aitken also states that it has been shown that the alkaloidal products formed by the putrefaction of albuminous substances vary according to the stage of decay at which they are produced. At first, the poisonous action of the ptomaines may be slight; but as decomposition advances the poisons become more virulent, while after a still longer period they become more broken up and lose to a greater extent their poisonous power. (Brunton.)

"In addition to these alkaloids obtained by Brieger, a number of poisons have been got by other workers from decomposing articles of food, or from dead bodies, and even from portions of healthy animal bodies, and although these may not have been obtained in the same state of purity, may not have had their chemic constituents so well defined as Brieger's, they are still as unknown extractives, x, y, z's of great interest and importance."

Experimentation has even shown that the primary products of albuminous decomposition of digestive ferments, such as peptones, are poisonous; and that pepsin will split up albuminous substances still further; and Dr. Brunton very aptly sounds the tocsin of caution against the extreme and indiscriminate use of various digestive ferments, and of the many varied artificially digested foods which have now become common. Consequently, it behooves us to study the products of albuminous decompositions as a matter of much practical importance, not only as regards pathology, but as regards therapeutics. Now it would not be possible to exclude these animal alkaloids from the general economy, for Gautier has proven that they are a necessary product of *vital physiologic processes.* His experiments show "that about four-fifths of our disassimilations are the result of transformations within the body, comparable to the oxidation of alcohol, and that the remaining one-fifth of the disassimilations are formed at the expense of the living tissues themselves, free of all demands on foreign oxygen." In other words, "a fifth part of our tissues live after the manner of ferments, that is they are anerobious or putrefactive as to their life." "Hence the possibility of alkaloids being thus formed within the living organism, independent of bacterial fermentation is quite within our conception."

Bio-chemically, the same author states that much has been proved, and he does not doubt that poisonous alkaloids are continuously formed in healthy men and animals by the decomposition of albumen in the intestinal canal, during the process of digestion, or in the blood and tissues generally by the metabolism which occurs during the functional activities of life. He believes that a considerable production of alkaloids takes place in the intestines, both when the digestive processes are normal, and more especially when they are disordered. Were all the alkaloids to remain within the body, poisoning would undoubtedly ensue, and Bouchard makes the statement that the alkaloids formed in the intestines of a healthy man in twenty-four hours would be sufficient to kill him if they were all absorbed and secretion stopped. Another statement, made by the same author, tends to show the power of these agents as an etiologic factor, and which at this time will probably not be questioned: "That the nervous disturbance which occurs in cases of dyspepsia is due to poisoning by ptomaines;" "that they augment notably in the course of certain maladies—in typhoid fever for instance."

[*To be Continued.*]

